# Encapsulation of Pharmaceutical and Nutraceutical Active Ingredients Using Electrospinning Processes

**DOI:** 10.3390/nano11081968

**Published:** 2021-07-30

**Authors:** Mina Zare, Karolina Dziemidowicz, Gareth R. Williams, Seeram Ramakrishna

**Affiliations:** 1 Center for Nanotechnology and Sustainability, National University of Singapore, Singapore 117581, Singapore; 2 UCL School of Pharmacy, University College London, 29-39 Brunswick Square, London WC1N 1AX, UK; k.dziemidowicz@ucl.ac.uk

**Keywords:** electrospinning, encapsulation, nanocarrier, nanofiber, nutraceuticals, probiotics, target drug delivery

## Abstract

Electrospinning is an inexpensive and powerful method that employs a polymer solution and strong electric field to produce nanofibers. These can be applied in diverse biological and medical applications. Due to their large surface area, controllable surface functionalization and properties, and typically high biocompatibility electrospun nanofibers are recognized as promising materials for the manufacturing of drug delivery systems. Electrospinning offers the potential to formulate poorly soluble drugs as amorphous solid dispersions to improve solubility, bioavailability and targeting of drug release. It is also a successful strategy for the encapsulation of nutraceuticals. This review aims to briefly discuss the concept of electrospinning and recent progress in manufacturing electrospun drug delivery systems. It will further consider in detail the encapsulation of nutraceuticals, particularly probiotics.

## 1. Introduction

**Figure 1:**
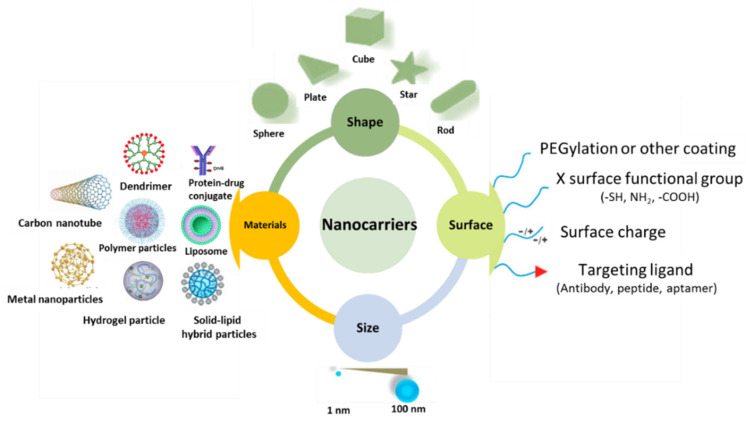
Nanocarriers for drug delivery and their biophysiochemical properties. Many factors are involving in determining their therapeutic potential including size, shape, materials, and surface chemistry.

Nanotechnology has great promise for the prevention, diagnosis, and treatment of disease [1]. Drug delivery using nanocarriers has received particularly extensive attention (Figure 1) [2]. Nanocarriers can help to minimize the side effects of drugs, and enhance the therapeutic efficacy and targeting precision [3,4,5]. Liposomes [6], nanoemulsions [7,8], Pickering emulsions [9], micelles [10,11], dendrimers [12], and polymeric nanoparticles have all been widely explored as carriers for drug delivery systems [3], but despite the significant body of work carried out there remain many challenges to overcome, particularly in terms of solubility and the accuracy of targeting [13,14].

The drug release from the delivery carrier system can be controlled by diffusion, degradation, swelling, and affinity-based mechanisms. The balance and rate of these is a function of the materials from which the carrier is constructed. Polymers in particular offer the opportunity to tune the release rate over a wide range. Synthethic and natural polymers are both widely available, and many are biodegradable and biocompatible [15].

The electrohydrodynamic (EHD) technique is a material fabrication method in which a polymer solution is dispersed into a fine jet under the influence of an electric field. This results in the formation of fibers (electrospinning) or particles (electrospraying). The main difference between electrospinning and electrospraying is the solution viscosity: electrospray uses a less viscous polymer solution, while at higher viscosities electrospinning occurs [16]. EHD is a low-cost, time-effective and versatile method has been used to process a wide range of pharmaceutically relevant materials into polymer carriers [17]. Polymer-based electrospun fibers loaded with therapeutic agents ranging from small molecules [18] to proteins [19] and bacteria [20] have shown both sustained and localised drug release in preclinical models. Moreover, electrospun nanofibers have potential as biomaterials for tissue engineering applications due to their tunable mechanical and handling properties, large surface area, and a 3D structure that mimics the extracellular matrix [21,22].

Key advantages of electrospinning include (i) the ability to process diverse polymers; (ii) submicron diameters are easily attained; (iii) portable systems are available; (iv) generation of a 3D fibrous structure. Disadvantages include (i) potential issues with solvent removal; (ii) and typically low throughput rates [23]. The fabrication rate of laboratory-scale electrospinning is usually in the range of 0.01–1 g/h, [24], much lower than pharmaceutical industry requirements. To resolve this issue, a number of companies have developed technological solutions for large-scale production [25]. As a result, it is now possible to produce electrospun materials on the tonnes p.a. scale under Good Manufacturing Practice conditions.

In this review, we will discuss the principles of electrospinning and the fabrication of electrospun fibers in the context of biomedical applications. Because the latter are well explored, they allow us to illustrate the power of the electrospinning approach. We will then focus in detail on the nascent field of using electrospinning for encapsulation of nutraceuticals within polymer nanofibers, which again shows great promise.

## 2. Principles of Electrospinning

The main components of the EHD apparatus include a high-voltage power supply, a precision syringe pump, a syringe loaded with a polymer solution and fitted with a conductive metal needle (the spinneret) and a collector. To maintain an electric field, the power supply is connected to both the spinneret and the collector. The polymer solution is extruded through the charged spinneret, with the syringe pump ensuring a controlled flow rate. Without the application of electric charge, the polymer solution exits the needle forming a spherical droplet owing to the surface tension forces [14]. When subjected to high voltage during extrusion through a metal needle, the liquid surface becomes charged, causing the spherical droplet to be retained at the capillary tip. With sufficient voltage applied, the meniscus deforms into a conical structure, which is often referred to as the Taylor cone [26]. In electrospinning, a polymer jet is emitted at the tip of the Taylor cone, and this then stretches and reduces in diameter as it travels towards the collector. The solvent present in the polymer solution evaporates as the jet is drawn and accelerates towards the collector, therefore producing a solid fibrous product [14].

Although the assembly of the EHD apparatus and product collection is relatively simple, the optimisation of experimental parameters necessary for the fabrication of uniform and reproducible scaffolds requires extensive and detailed experimentation. Critical variables can be broadly classified into solution properties and processing parameters. Key solution properties include the nature of the polymer(s) to be processed, their concentration and molecular weight, and solvent volatility. These all influence the conductivity and viscosity of the solution, and thus impact its spinnability.

Several aspects need to be considered when choosing the polymer carrier for the electrospinning solution. Probably the most important consideration is the intended application of the product. The polymer degradation half-life and by-products, biocompatibility, and solubility will heavily influence the potential applications of the product. For example, for fast-release applications, a polymer with a relatively rapid dissolution/degradation rate and high solubility in aqueous solvents (such as polyvinylpyrrolidone (PVP)) would be preferred [14]. In contrast, when designing a long-term surgical implant, a hydrophobic polymer with slow degradation rates would be more suitable. Many biodegradable synthetic polymers have been explored in electrospinning, including poly(ε-caprolactone) (PCL), polylactic-co-glycolic acid (PLGA), and polylactic acid (PLA) [27,28]. In some cases, polymers with special characteristics such as thermo- or pH-sensitivity are of interest, aiding targeted delivery to a chosen site [29]. Probably the most investigated stimuli-responsive polymers include poly(N-iopropylacrylamide), which has thermoresponsive properties, as well as the polymethacrylate family of polymers and poly(4-vinylpyridine), which are pH-sensitive [30,31,32].

The factors affecting the electrospinning process can be divided into (i) processing, (ii) solution and solvent, and (iii) environment parameters (Table 1) [15,33,34,35]. Important processing parameters include the flow rate, applied voltage, and the distance between the collector and the spinneret. The latter two determine the electric field strength, and typically we would work at a distance of 5–20 cm and applied voltage of 5–35 kV. These factors, together with the flow rate, will affect the stability of the spinning process and the diameter of the resultant fibers [23,32]. The solution parameters include the solvent, polymer concentration, viscosity and solution conductivity. Environmental parameters (temperature and relative humidity) also need to be taken into consideration [36].

### 2.1. Electrospinning Methods

**Figure 2:**
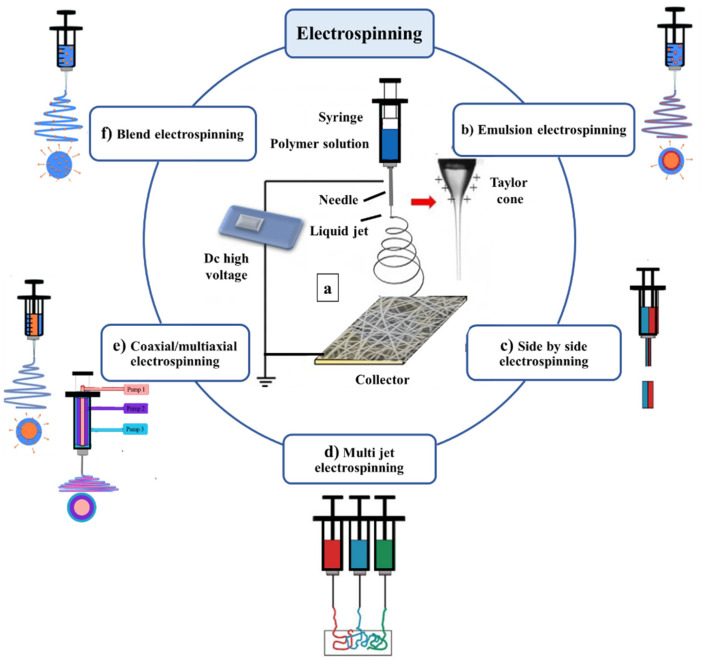
Schematic of the different routes to drug incorporation into a polymer carrier through electrospinning.

**Figure 3:**
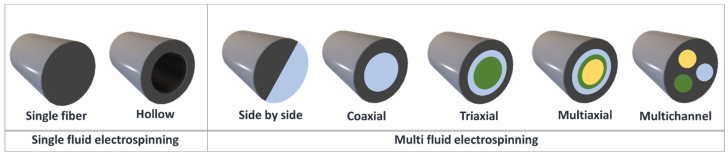
The cross-sections of fibers generated using the various electrospinning approaches.

There are several electrospinning approaches that can be applied for the incorporation of drugs into polymer carriers. These include blend electrospinning, emulsion electrospinning, side by side electrospinning (yielding Janus products), multi-jet electrospinning, and coaxial/multiaxial electrospinning, which result in multilayer structures (see Figure 2). It is also possible to surface functionalise the fibers after spinning. The cross-sections of the resultant nanofibers are illustrated in Figure 3.

### 2.2. Single Fluid Electrospinning

#### 2.2.1. Blend Electrospinning

In this technique, the drug and the polymer carrier are dissolved in a suitable solvent to form a homogenous spinning solution. This approach can yield a wide range of drug release profiles, from very rapid release (in seconds) to sustained release over weeks or months. [37,38]. The main weakness of this approach is the commonly observed burst release phenomenon [39]. This arises because the surface area to volume ratio of the fibers is very high, and thus a large amount of drug is present at the surface. This can easily diffuse into solution, while the drug at the center of the fiber takes longer to escape. As a result, first-order release profiles are commonly observed. Further, the drug loading which can be achieved may be limited, as it can be challenging to identify a suitable solvent in which both the drug and polymer are soluble [40].

#### 2.2.2. Emulsion Electrospinning

Emulsion electrospinning fabricates core–shell nanofibers using an emulsion for spinning. This can be beneficial for the encapsulation of growth factors, proteins, and drugs in the core of the product. Three key components are required to form a stable emulsion: (a) an oil phase, (b) a water phase, and (c) surfactants/emulsifiers. These also impact the drug release properties. Typically, a hydrophobic polymer is dissolved in an organic solvent (oil phase), while hydrophilic drugs are dispersed in water. For instance, Tao, et al. in 2020 manufactured polycaprolactone/carboxymethyl chitosan (CS)/sodium alginate fibers by emulsion electrospinning with minimal use of organic solvents, which had a positive impact on osteoblast viability and osteogenesis g [41]. In other work, a reservoir-type system comprising PLA/theophylline was fabricated via emulsion electrospinning, showing that nanofibers can be prepared to incorporate a water-soluble drug in a hydrophobic polymer. The core/shell structure was able to prevent any burst release [42].

### 2.3. Multi-Fluid Electrospinning

#### 2.3.1. Multi-Jet Electrospinning

Multi-jet electrospinning exists in two forms: needleless and needle-based. It is beneficial for large-scale nanofiber fabrication since it can significantly increase throughput. It also affords the opportunity to prepare multicomponent fiber mats, with multiple populations of fibers made from different materials integrated into the same scaffold. This can be useful when it is desirable to have multiple polymers in a formulation but they cannot be dissolved in the same solution. The resultant fiber mat can deliver multiple drugs at varied rates, and the different fiber populations can also influence the mechanical and cell adhesion properties. The drawback of multi-jet spinning in the needle modality is that the electric fields around the different needles interact with one another, which can cause spinning to be erratic. It is also difficult to calculate the optimal arrangement of needles. These issues can be ameliorated by using a needleless process, or employing secondary or auxiliary electrodes [43,44,45,46].

#### 2.3.2. Side by Side Electrospinning

In this approach, multiple spinning solutions are fed through separate spinnerets placed next to each other. The key advantage of this approach is the side-by-side Janus morphology of the resultant materials, which allows for the direct contact of both compartments with the biological microenvironment [47,48]. The spinneret design and careful optimization of electrospinning parameters are critical to the success of this method. One example of this was reported by Zheng et al. in 2021. Tamoxifen was included as a chemotherapeutic drug, and PVP and ethyl cellulose (EC) were used as the polymer matrices. Zheng’s study revealed that shape, structure, and composition are clearly all critical elements for designing functional nanomaterials [49].

#### 2.3.3. Coaxial/Multiaxial Electrospinning

Coaxial electrospinning features a concentrically aligned dual nozzle. This results in core–shell fibers, which can have beneficial properties [50] and advantages over blend and emulsion techniques (e.g., overcoming the burst release commonly seen with monolithic fibers from blend spinning) [16]. In coaxial EHD, two fluids are dispensed simultaneously. The core solution is pumped through an inner needle and the shell solution through an outer needle. This technique is often used for encapsulation of labile biomolecules such as protein active ingredients [21,51], employing an organic solvent for the polymer shell solution and an aqueous solution of protein as the core. As both solutions are physically separated until the formation of the fiber, protein exposure to organic solvents can be limited and accidental degradation minimised. The benefits of using coaxial EHDA to process protein active ingredients were recently reviewed in detail by Moreira et al. [16]. The coaxial electrospinning approach can also be used to slow down the release rate of small drug molecules from a hydrophobic matrix or to encapsulate a liquid in the core. For instance, Baykara and Taylan employed coaxial electrospinning to generate antimicrobial PVA (shell)/Nigella sativa seed oil (core) fibers [52]. It is also possible to prepare multilayer fibers using triaxial spinning (three fluids). Liu et al. used the triaxial electrospinning technique to encapsulate ferulic acid in cellulose acetate nanofibers. An in vitro study showed almost zero-order release [53]. Quad-axial nanofibers (generated by processing four fluids simultaneously) can further be prepared; Zhang et al. employed polycaprolactone and gelatin for encapsulation of the antimicrobial moxifloxacin [54].

### 2.4. Electrospun Drug Delivery Systems

There is a very significant body of literature reporting the use of electrospun fibers for drug delivery. Some examples are discussed above, and a further (non-exhaustive) selection of representative examples is given in Table 2.

## 3. Encapsulation of Nutraceuticals by Electrospinning

### 3.1. Nutraceuticals

Nutraceuticals are food-derived supplements that are potentially beneficial in the prevention and treatment of disease. They include probiotics (living bacteria thought to positively affect health), prebiotics (compounds that promote the growth of beneficial microorganisms), omega-3 and structured lipids, phytochemicals and plant extracts, carbohydrates, carotenoids and antioxidants, amino acids, peptides, and proteins, vitamins, and minerals. A brief summary is given in Table 3. Precise strategies are needed for nutraceutical delivery to aid in protecting sensitive moieties from stress conditions during processing, prevent unwanted interactions between the nutraceuticals and food matrix, and obviate degradation before release at the target site. These challenges can be overcome through encapsulation [77,78]. Food products such as meat (fermented sausages), dairy (cheese and yogurt), juices (from fruits and vegetables), bakery products (biscuits, cakes, bread), and others (fermented beverages, mayonnaise, ice cream) can all be functionalized by adding probiotic microcapsules [79].

Considering some of the examples from Table 3, probiotics such as *Lactobacillus plantarum*, *Lactobacillus* sp., *Lactobacillus casei*, and *Bifidobacterium* are naturally present in yogurt, cheese, and fermented milk. These can have a number of benefits. *Lactobacillus* sp. and *Lactobacillus casei* aid in the removal of cholesterol, and possess activity against cancer cell proliferation, as well as in reducing the risk of osteoporosis [80]. They can thus effectively be used for the development of functional foods. Minerals are present in animal meat, plant products and milk products, and are important for the treatment of many diseases such as anemia and osteoporosis [81]. Carotenoids are present in most fruits and vegetables, plants, algae, and photosynthetic bacteria. They are reported to have a range of benefits in eye health, cognitive function and cardiovascular health, and also there are possible benefits in preventing some types of cancer [82]. Importantly, humans cannot synthesize carotenoids, and thus must obtain them from food.

### 3.2. Small Molecule Nutraceuticals

Many nutraceuticals comprise small molecules. The challenges in delivering these often mirror those encountered with drugs and detailed in Section 3.1. Such bioactive compounds (e.g., vitamins, essential oils) can have antimicrobial, antioxidant, antifungal, and antiseptic properties [90,91,92,93]. However, they tend also to suffer from low aqueous solubility, which can be overcome by electrospinning nanofibers. In the context of nutraceuticals, one approach which has attracted particular attention is to generate fibers from inclusion complexes of the compound of interest with a cyclodextrin (CD). Fibers can be prepared either using the inclusion complex and a polymer, or from highly concentrated solutions of the inclusion complex alone [94,95]. A number of studies report the successful electrospinning of fast dissolving fibers comprising inclusion complexes of curcumin [96], ferulic acid [97], and α-lipoic acid [98], among others. Fiber formation can also help to overcome issues other than solubility. For instance, α-lipoic acid is a natural antioxidant with low solubility and poor thermal and oxidative stability. Electrospun nanofibers of α-lipoic acid–CD complexes can help to retain their antioxidant properties, as well as accelerate the dissolution rate [98]. Similar results have been reported for vitamin E, where fibers prepared from CD inclusion complexes could prolong the shelf life and increase the photostability.

### 3.3. Pre- and Probiotics

There are well-known beneficial interactions between the bacteria in the gut (the microbiota) and the human body. The manner in which bacteria contained within the gut “talk” to the immune system is of great importance to human health, and probiotics and nutraceuticals can play a major role in improving this [99,100]. Nutraceuticals and probiotics can for instance cause a significant reduction in insulin resistance, improve the level of glucose in the blood, lower the prevalence of obesity, and reduce total and visceral adipose tissue (VAT) weight [101,102]. As a result, both probiotics and prebiotics have attracted significant research attention [100,103].

Interest in the human microbiota has grown considerably in recent years, with a wide range of probiotic products available on the market. While most probiotics are living microorganisms that confer health benefits to the host, it has been shown that dead bacteria and their components can also exhibit probiotic properties [104]. Probiotics are offered in a variety of delivery systems ranging from capsules or biopolymeric gel matrices to food products such as yoghurts [105,106]. Studies on probiotics have demonstrated they can lead to an enhancement in intestinal epithelial integrity, regulation of the immune system in the gastrointestinal tract, protection from gut barrier disruption, and inhibition of the growth of pathogenic bacteria [107,108,109]. Research has further revealed the positive impact of ingesting these types of organisms on the alleviation of symptoms associated with irritable bowel syndrome (IBS), counteracting antibiotic-induced diarrhea, obesity and obesity-related disorders in glucose metabolism, and ulcerative colitis [103,109,110,111,112]. Probiotics are additionally reported to have health benefits beyond the gastrointestinal tract, including for cancer, diabetes, human immunodeficiency virus infection [111,112], central nervous system disorders, cardiovascular diseases, and liver disease [107,108]. In December 2020, there were 245 registered clinical trials exploring the effect of prebiotics (with or without probiotics) on autism, colic, colon cancer, aging, atopic dermatitis, infant growth, obesity, bariatric surgery, constipation and diarrhea, and IBS; this clearly demonstrates significant investment and potential for a range of healthcare applications [103].

**Figure 4:**
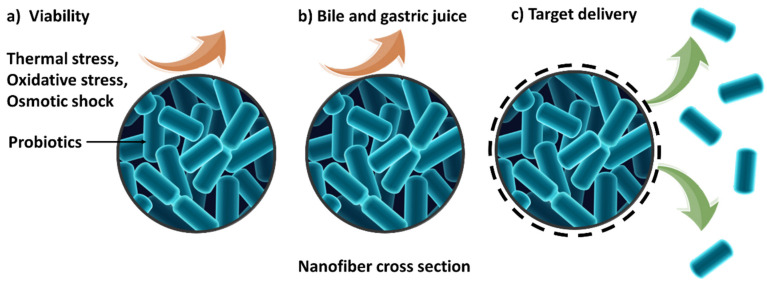
A schematic for the use of electrospun nanofibers in probiotic delivery. Encapsulation in a polymer matrix can (**a**) protect the organisms from external stresses and thus maintain viability during manufacturing and storage, (**b**) protect the probiotics from the bile and stomach acids, and (**c**) permit dissolution of the formulation and release of viable probiotics at the target site.

Probiotics are living microorganisms, which makes them particularly challenging to safely process and deliver to the target site. They need to be viable upon arrival in order to elicit therapeutic responses, and so great care must be taken during formulation to prevent their accidental death. There is a range of pharmaceutical technologies which could be considered for probiotic encapsulation, including spray drying, hot-melt extrusion, spray-freeze drying, freeze-drying, and coacervation [113]. Many of these methods require harsh conditions (e.g., heat in spray drying or hot melt extrusion) that can damage probiotics during manufacturing. Since electrospinning does not require the use of any heat, it has great potential in this field and offers advantages over more traditional pharmaceutical technologies. It should be noted in addition that, once the manufacturing hurdles are overcome, probiotics still need to reach the lower parts of the intestinal tract to have the desired therapeutic effect. To be effective they must be metabolically active, and therefore able to survive storage, transport, digestive enzymes (lipase, protease, amylase), mineral ions, stomach acids (pH 1–3) and bile during gastrointestinal tract transit [111,112,114]. The ability of electrospun fibers to overcome some of these challenges is depicted schematically in Figure 4.

A range of probiotic microorganisms has been integrated into electrospun nanofibers with the ultimate aim of reaching these goals [115]. These studies are summarized in Table 4. Commonly explored probiotic strains are *Bifidobacterium bifidum*, *Bifidobacterium breve*, *Lactobacillus acidophilus,* and *Lactobacillus rhamnosus* [111]. For instance, Mojaveri et al. fabricated a nanofiber mat using a PVA/CS blend and loaded it with both the probiotic *Bifidobacterium animalis* and inulin as a prebiotic. The authors found that electrospinning was a promising approach for the protection of living probiotics and functional food products [116].

In general, the viability of probiotics is found to be high after electrospinning both when the fibers are stored at room temperature and in the refrigerator [117]. Electrospun fibers can possess high loading capacities for probiotics, and could lead to formulations able to yield local delivery to re-establish the microbiota balance, e.g., in the vagina or intestine [118]. While most studies have focused on simple blend electrospinning, coaxial electrospinning has also recently been explored for delivery of probiotics, again showing that the encapsulated probiotics have improved thermal stability and are able to resist harsh conditions [119]. In this study, the monolithic fibers from blend spinning were found to be unable to protect the probiotics from damage in the acidic conditions of the stomach, and almost all the cells lost their viability. In contrast, coaxial electrospinning could provide better protection and controlled release [119,120]. In vivo studies using multi-layer fiber mats encapsulating *Bacillus coagulans* using CS/alginate/CS/alginate found that this strategy protects probiotics against gastrointestinal tract insults and improves their adhesion and growth in the intestine [121]. In contrast, single layer CS alone did not provide benefits against simulated gastric fluid and bile insults in vitro [121]. Overall, it is clear that electrospinning circumvents the common drawbacks of probiotic degradation within a formulation, and preserves biological action after complete release from polymer fiber [122]. While some studies suggest that simple blend electrospinning is sufficient to provide these advantages, others indicate that a coaxial or multilayer is more effective at protecting the incorporated probiotics.

## 4. Conclusions, Challenges, and Future Perspectives

Recent progress clearly indicates the great potential of electrohydrodynamic processes in the fabrication of nanofibers for pharmaceutical applications. As a versatile and highly tunable nanomaterial fabrication technology, electrospinning can be used to encapsulate a wide array of therapeutic agents, with most attention having been devoted to working with pharmaceutical active ingredients ranging from small molecules to proteins. In addition, however, there are significant opportunities for the delivery of nutraceutical molecules. Challenges of low solubility and stability for nutraceutical small molecules can be overcome by preparing electrospun formulations, with cyclodextrin inclusion complex fibers having been shown to be particularly promising here. In the latter context, electrospun fibers are found to lead to improved probiotic viability, their ability to resist harsh conditions (e.g., heat) commonly used in food processing, improved storage stability, and the potential to localise delivery to the target site in the lower parts of the gastrointestinal tract. There remain challenges to be overcome, however, and it will be necessary to perform significant amounts of additional in vivo work and clinical trials to fully validate the potential of such electrospun formulations. In addition, methods by which the fiber formulations could be incorporated into food processing pathways will need careful attention. However, these obstacles are clearly surmountable: the pharmaceutical industry has extensive experience of this, and significantly more complex formulations than those from electrospinning have already made it to the clinic. To date, there are no commercial pharmaceutical or nutraceutical products from electrospinning on the market, but there are formulations in stage II clinical trials and the direction of travel is very positive. Given the huge recent advances which have been made in the scale-up of electrospinning, the authors are confident that in the next 10 years, we will see both pharmaceutical and nutraceutical products from EHDA enter the market.

## Figures and Tables

**Table 1:** Key factors affecting the electrospinning process.

Parameter	Effect
**Processing parameters**	
Flow rate	↑ Flow rate leads to ↑ fiber diameter and ultimately unstable Taylor cone
Voltage	↑ Fiber diameter decrease, ↓ no fiber formation
Collector	Type of collector impacts 3D structure and fiber alignment
Distance needle-collector	↑ Non-uniform beaded fibers are formed, ↓ no fiber formation
**Solvent parameters**	
Dielectric constant	↑ Fiber diameter decreases, ↓ beaded fibers are formed
Volatility	↑ High porosity and surface area, ↓ difficult to remove solvent
**Solution parameters**	
Viscosity	↑ Thicker and continuous nanofibers. If too high, beads and nozzle clogging are observed. ↓ Finer nanofibers, but if viscosity too low then electrospraying will result
Concentration	↑ Fiber formation with higher diameter and fewer beads. If too high nozzle clogging can be observed. ↓ If too low sputtering can happen
**Environmental parameters**	
Humidity	Humidity impacts solvent evaporation rate. ↑ Humidity can led to incomplete drying
Temperature	Temperature impacts viscosity and solvent evaporation rate. ↑ Temperature led to ↓ viscosity and more efficient evaporation of solvent.

**Table 2:** Exemplar drug delivery applications of electrospun nanocarriers.

Polymer Carrier	Drug	Indications	Ref.
Polylactic acid (PLA)	Dichloroacetate	Antineoplastic	[55]
PLA	Doxorubicin/doxorubicin hydrochloride (Dox-HCl)	Antineoplastic	[56]
PLA, polyethylene oxide (PEO)	Rapamycin	Antineoplastic	[57]
Polycaprolactone (PCL)	Naproxen	Anti-inflammatory	[58]
PCL	Metronidazole/ciprofloxacin	Antimicrobial	[59]
PCL	Ibuprofen Carvedilol	Anti-inflammatory Beta blocker	[60]
PCL	Paclitaxel	Antineoplastic	[61]
PCL	Gentamicin/Ag	Antimicrobial	[62]
PCL, gelatin	Doxorubicin (Dox)	Antineoplastic	[63]
PCL, gelatin	Ketoprofen	Anti-inflammatory	[64]
PCL, Polyethylene glycol (PEG)	Curcumin/doxorubicin	Antineoplastic	[65]
PCL, Polyvinylpyrrolidone (PVP), Cellulose acetate (CA)	Nisin	Antimicrobial	[66]
PCL, Chitosan (CS)	Ciprofloxacin	Antimicrobial	[67]
CS, PEO	Insulin	Transbuccal insulin delivery	[68]
PVP	Carvedilol	Buccal delivery	[69]
CA, PVP	Amoxicillin	Antimicrobial	[70]
PEG, Polylactic-co-Glycolic Acid (PLGA)	10-Hydroxycamptothecin/hydrophilic tea polyphenol	Antineoplastic	[71]
PLGA	Growth factors	Regenerative medicine	[72]
PVP, hyperbranched poly(butylene adipate (HB)	Artemisinin	Antineoplastic	[73]
Ethyl cellulose (EC), zein	Indomethacin	Anti-inflammatory	[74]
Polycarbonate polyurethane (PCNU)	Antimicrobial oligomer	Antimicrobial	[75]
Polymethyl vinyl ether-*alt*-maleic ethyl monoester	Salicylic acid/methyl salicylate capsaicin	Psoriatic lesion treatment	[76]

**Table 3:** Selected nutraceuticals of interest to the food industry, and their associated health benefits.

Category	Food Source	Examples	Some Associated Health Benefits	Ref.
Probiotics	Yogurt, sourdough, kimchi, sauerkraut, organic whey, bread, milk, cheese	*Lactobacillus plantarum*, *Lactobacillus* sp., *Lactobacillus casei*, *Bifidobacterium*	Modulation of microbial signatures of health and disease, improved immune status and intestinal health	[83,84]
Bioactive peptides	Fish, meat, milk, plants	Peptides in milk, eggs, and sardines	Antihypertensive properties	[81]
Dietary lipids	Fish, flaxseed, canola, calamari, krill, algae, genetically modified plants and seeds	Alpha-linoleic acid, docosahexaenoic acid, eicosapentaenoic acid	Reduced risk of atherosclerosis Improved cardiovascular health Improved cognition and brain health Reduced risk of certain cancers	[78,85]
	Milk fat	Conjugated linolenic acid	Reduced risk of atherosclerosis Anticarcinogenic, immunomodulatory, and anti-inflammatory properties	[78,86]
Vitamins	Fruits, dairy products, vegetables and meat	Vitamin A, C, D, E, K, B_1_, B_3_, B_6_, B_9_, B_12_	Range of health benefits, (e.g., vitamin A/C/E are antioxidants, vitamin K is essential for clotting of blood)	[78,87]
Minerals	Usually available as salts	Zinc, calcium, iron, magnesium, phosphorus	Range of health benefits (e.g., zinc essential for cell reproduction)	[78,87]
Phenolic compounds and polyphenols	Wine, olives, tea, pomegranates, cocoa, vegetables, grapeseed, grapes, seeds	Flavones, flavanols, catechins, curcuminoids, resveratrol phenolic acids	Reduced oxidative stress Protection against cardiovascular, neurodegenerative, and metabolic diseases and cancer	[78,88]
Carotenoids	Green leafy vegetables, microalgae, marigolds, carrots, tomatoes	Astaxanthin, lutein, lycopene, β-carotene	Protection against cancer, heart disease, and age-related macular degeneration, age-related macular eye disease and cataracts	[78,89]

**Table 4:** A list of probiotics that have been incorporated into electrospun nanofibers, along with the potential applications.

Polymer	Bacterium	Source Code/Strain	Type of Electrospinning	Purpose	Ref.
PVA/CA	*E. coli*	EcN1917	Dual/multi-nozzle	Delivery system enhancing viability in the gastrointestinal tract, and storage stability	[123]
Alginate, PEO, polysorbate 80	*E. coli*	K12 MG1655	Blend	Biocompatible, edible delivery system targeted to the gut	[20]
Eudragit^®^ L100, sodium alginate	*Lb. paracasei*		Blend	Controlled release of probiotics, and pH-targeted release.	[122]
Alginate	*Lb. paracasei*	KS-199	Blend	Increased viability of probiotic cargo	[124]
PEO	*Lb. plantarum*	ATCC 8014	Blend	High loading and long-term viability; local delivery to re-establish the microbiota balance, e.g., in vagina	[118]
Carboxymethyl cellulose/PEO	*S. epidermidis*	BH1	Blend	Potential preventive treatment of the diabetic foot	[125]
PEO/CS	*Bacillus* sp.	25.2.M	Blend	Periodontal disease	[126]
Fructo-oligosaccharides, PVA	*Lb. plantarum*		Blend	Improvement of probiotic viability and thermal stability	[120]
PVA	*B. animalis*	Bb12	Blend	Increased viability on storage	[117]
PEO	*Lb. plantarum*	423	Blend	Bacteriocin and probiotic delivery system	[127]
	*Enterococcus faecium*	HKLHS			
Soluble dietary fiber, oil-palm trunk, oil-palm fronds, PVA	*Lb. acidophilus*	FTDC 8933	Blend	Soluble dietary fiber, thermal protection of probiotics in heat-processed foods, improved viability on storage	[128]
PVA, PVP	*Lb. acidophilus*		Blend	Bacterial vaginosis	[129]
*CS, PVA, INU*	*B. animalis*	*lactis* Bb12	Blend	Delivery system	[116]
Sodium alginate, PVA	*L. plantarum*		Coaxial	Improved thermal stability, ability to resist harsh conditions.	[119]
